# *N*-{3-[2-(4-Fluoro­phen­oxy)eth­yl]-2,4-dioxo-1,3-diaza­spiro­[4.5]decan-7-yl}-4-meth­oxy­benzene­sulfonamide

**DOI:** 10.1107/S1600536811053980

**Published:** 2011-12-21

**Authors:** M. Vinduvahini, S. Jeyaseelan, J. Shylajakumari, H. D. Revanasiddappa, Venkatesh B. Devaru

**Affiliations:** a Department of Physics, Sri D Devaraja Urs Govt. First Grade College, Hunsur 571 105, Mysore District, Karnataka, India; b Department of Physics, Yuvaraja’s College (Constituent College), University of Mysore, Mysore 570 005, Karnataka, India; c Department of Physics, AVK College for Women, Hassan 573 201, Karnataka, India; d Department of Studies in Chemistry, Manasagangotri, University of Mysore, Mysore 570 006, Karnataka, India; e Department of P.G. Studies in Physics, L V D College, Raichur 584 103, Karnataka, India

## Abstract

In the title compound, C_23_H_26_FN_3_O_6_S, the two terminal aromatic rings form a dihedral angle of 49.26 (12)°. The cyclo­hexane ring adopts a chair conformation and the five-membered ring is essentially planar, with a maximum deviation from planarity of 0.0456 (19) Å. The dihedral angles between the five-membered ring and the meth­oxy­benzene and fluoro­benzene rings are 33.56 (11) and 81.94 (12)°, respectively. The crystal structure displays N—H⋯O hydrogen bonds as well as weak inter­molecular C—H⋯O inter­actions.

## Related literature

For the biological activity of related compounds, see: Cartwright *et al.* (2007 ▶); Collins (2000 ▶); Warshakoon *et al.* (2006 ▶) and for their pharmaceutical activity, see: Kiselyov *et al.* (2006 ▶); Sakthivel & Cook (2005 ▶); Eldrup *et al.* (2004 ▶); Bamford *et al.* (2005 ▶); Puerstinger *et al.* (2006 ▶).
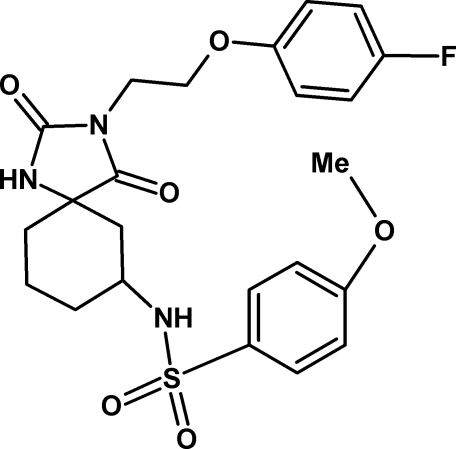


## Experimental

### 

#### Crystal data

C_23_H_26_FN_3_O_6_S
*M* *_r_* = 491.53
Monoclinic, 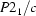

*a* = 11.926 (5) Å
*b* = 11.025 (5) Å
*c* = 18.508 (5) Å
β = 97.271 (5)°
*V* = 2413.9 (16) Å^3^
*Z* = 4
Mo *K*α radiation
μ = 0.19 mm^−1^
*T* = 293 K
0.20 × 0.15 × 0.12 mm

#### Data collection

Oxford Diffraction Xcalibur diffractometer
Absorption correction: multi-scan (*CrysAlis PRO RED*; Oxford Diffraction, 2010 ▶) *T*_min_ = 0.771, *T*_max_ = 1.000
21931 measured reflections
4240 independent reflections
3435 reflections with *I* > 2σ(*I*)
*R*_int_ = 0.035

#### Refinement

*R*[*F* ^2^ > 2σ(*F* ^2^)] = 0.041
*wR*(*F* ^2^) = 0.121
*S* = 1.04
4240 reflections
307 parameters
H-atom parameters constrained
Δρ_max_ = 0.48 e Å^−3^
Δρ_min_ = −0.50 e Å^−3^

### 

Data collection: *CrysAlis PRO CCD* (Oxford Diffraction, 2010 ▶); cell refinement: *CrysAlis PRO CCD*; data reduction: *CrysAlis PRO RED* (Oxford Diffraction, 2010 ▶); program(s) used to solve structure: *SHELXS97* (Sheldrick, 2008 ▶); program(s) used to refine structure: *SHELXL97* (Sheldrick, 2008 ▶); molecular graphics: *XP* in *SHELXTL* (Sheldrick, 2008 ▶); software used to prepare material for publication: *WinGX* (Farrugia, 1999 ▶).

## Supplementary Material

Crystal structure: contains datablock(s) I, global. DOI: 10.1107/S1600536811053980/pk2365sup1.cif

Structure factors: contains datablock(s) I. DOI: 10.1107/S1600536811053980/pk2365Isup2.hkl

Supplementary material file. DOI: 10.1107/S1600536811053980/pk2365Isup3.cml

Additional supplementary materials:  crystallographic information; 3D view; checkCIF report

## Figures and Tables

**Table 1:** Hydrogen-bond geometry (Å, °)

*D*—H⋯*A*	*D*—H	H⋯*A*	*D*⋯*A*	*D*—H⋯*A*
N10—H10⋯O5^i^	0.86	2.08	2.924 (2)	168
N11—H11⋯O6^ii^	0.86	2.39	2.991 (3)	127
C20—H20*A*⋯O4^iii^	0.97	2.51	3.377 (3)	148
C31—H31⋯O4^iv^	0.93	2.48	3.327 (3)	152

Symmetry codes: (i) 
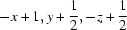
; (ii) 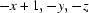; (iii) 
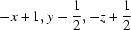
; (iv) 
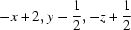
.

**Fig. 1.**
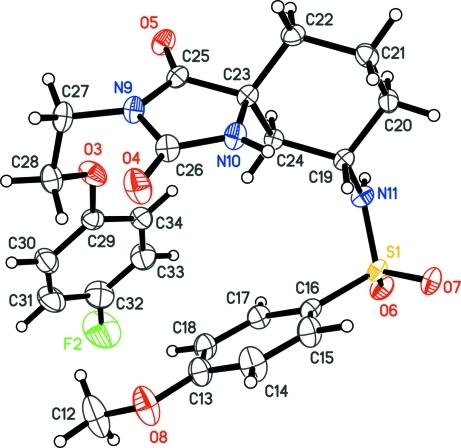
The title molecule with the displacement ellipsoids drawn at the 30% probability level. The H atoms are shown as spheres of arbitrary radii.

**Fig. 2.**
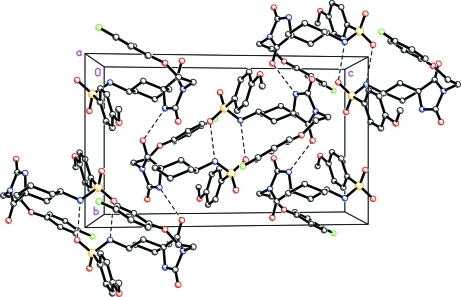
The packing of the molecules in the title compound, viewed down the a axis.

## supplementary crystallographic information

### Comment

One of the challenges of medicinal chemistry is the promotion of structural diversity, which can be achieved by the attachment of pharmacophoric groups to a given molecular scaffold using combinatorial chemistry. An example of such a process includes di- and tri-substituted hydantoins, which have been widely used in biological screenings, resulting in numerous pharmaceutical applications (Cartwright *et al.*, 2007; Collins, 2000; Warshakoon *et al.*,2006). Hydantion analogues have shown versatile therapeutic applications and some of them have been approved as drugs. For example, Fosphenytoin as a sodium channel antagonist is used for the treatment of epilepsy. Phenytoin has antiarrhythmic, anticonvulsant, and antineuralgic activities. Ethotoin and Mephenytoin both show anticonvulsant effects. Nilutamide is used in the treatment of prostate cancer (Kiselyov *et al.*, 2006; Sakthivel & Cook, 2005; Eldrup *et al.*, 2004; Bamford *et al.*, 2005; Puerstinger *et al.*, 2006).

The asymmetric unit of *N*-{3-[2-(4-fluorophenoxy)ethyl]-2,4-dioxo-1, 3-diazaspiro[4.5]dec-8-yl}-4-methoxy benzenesulfonamide, C_23_H_26_FN_3_O_6_S, contains just one molecule (Fig. 1). The two terminal benzene rings (C13···C18) and (C29···C34) form a dihedral angle of 49.26 (12)°. The cyclohexane (C19···C24) ring adopts a chair conformation, and the five-membered imidazolidine ring is essentially flat (max. deviation from mean plane = 0.0456 (19) Å). The dihedral angles between the five-membered ring and the methoxybenzene and fluorobenzene rings are 33.56 (11)° and 81.94 (12)°, respectively. The crystal structure displays intermolecular hydrogen bonds involving N10—H10···O5 and N11—H11···O6, as well as weak intermolecular C20—H20A···O4 and C31—H31···O4 interactions (Table 1). The packing of the molecules is depicted in Fig. 2.

### Experimental

A mixture of *tert*-butyl (4-oxocyclohexyl)carbamate (2 g, 9.37 mmol) and ammonium carbonate (1.08 g, 11.2 mmol) were taken in ethanol and water, respectively. A solution of sodium cyanide (2 g, 9.37 mmol) in water was added dropwise and the reaction mixture was stirred at RT for 24 hrs. A mixture of anhydrous potassium carbonate (1.28 g, 9.31 mmol) and 1-(2-bromoethoxy)-4-fluorobenzene (1.53 g, 6.9 mmol) in DMF (20 ml) was refluxed, and the solid was filtered, washed with water and dried in vacuum to give hydantoin. The *tert*-butyl dicarbonate (BOC) was de-protected using dioxane-HCl and it was basified to give the free amine. A mixture of the product (0.2 g, 0.622 mmol), triethylamine (0.075 g, 0.74 mmol) and sulfonyl chloride (0.115 g, 0.56 mmol) in dichloromethane (10 ml) was stirred at room temperature. After completion of the reaction (checked by TLC), the result was concentrated in vacuum to give the title compound (163 mg, 54%), which was recrystallized using 1:1 hexane: ethyl acetate as solvent.

### Refinement

All H atoms were positioned at calculated positions with N—H = 0.86°, C—H = 0.98° for methine, C—H = 0.97° for methylene H, C—H = 0.93° for aromatic H and C—H = 0.96° for methyl H and refined a riding model with *U_iso_(H)* = 1.5*U_eq_(C)* for methyl H and 1.2*U_eq_(C,N)* for the other hydrogen atoms.

### Crystal data

**Table:**

C_23_H_26_FN_3_O_6_S	*F*(000) = 1032
*M**_r_* = 491.53	*D*_x_ = 1.352 Mg m^−^^3^
Monoclinic, *P*2_1_/*c*	Melting point: 454 K
Hall symbol: -P 2ybc	Mo *K*α radiation, λ = 0.71073 Å
*a* = 11.926 (5) Å	Cell parameters from 4240 reflections
*b* = 11.025 (5) Å	θ = 2.2–25.0°
*c* = 18.508 (5) Å	µ = 0.19 mm^−^^1^
β = 97.271 (5)°	*T* = 293 K
*V* = 2413.9 (16) Å^3^	Prism, colourless
*Z* = 4	0.20 × 0.15 × 0.12 mm

### Data collection

**Table:**

Oxford Diffraction Xcalibur diffractometer	4240 independent reflections
Radiation source: fine-focus sealed tube	3435 reflections with *I* > 2σ(*I*)
graphite	*R*_int_ = 0.035
Detector resolution: 15.9821 pixels mm^-1^	θ_max_ = 25.0°, θ_min_ = 2.2°
ω scans	*h* = −14→14
Absorption correction: multi-scan (*CrysAlis PRO* RED; Oxford Diffraction, 2010)	*k* = −13→10
*T*_min_ = 0.771, *T*_max_ = 1.000	*l* = −20→22
21931 measured reflections	

### Refinement

**Table:**

Refinement on *F*^2^	Primary atom site location: structure-invariant direct methods
Least-squares matrix: full	Secondary atom site location: difference Fourier map
*R*[*F*^2^ > 2σ(*F*^2^)] = 0.041	Hydrogen site location: inferred from neighbouring sites
*wR*(*F*^2^) = 0.121	H-atom parameters constrained
*S* = 1.04	*w* = 1/[σ^2^(*F*_o_^2^) + (0.0687*P*)^2^ + 0.7995*P*] where *P* = (*F*_o_^2^ + 2*F*_c_^2^)/3
4240 reflections	(Δ/σ)_max_ < 0.001
307 parameters	Δρ_max_ = 0.48 e Å^−^^3^
0 restraints	Δρ_min_ = −0.50 e Å^−^^3^

### Special details

**Table:**

Experimental. *CrysAlis PRO*, Oxford Diffraction Ltd. Empirical absorption correction using spherical harmonics, implemented in SCALE3 ABSPACK scaling algorithm.Colourless solid: Yield: 103 mg, 67%); mp: 454k; IR cm^-1^ (KBr) 3359 (N—H), 1340 (S=O); Anal. Calcd For C_23_H_26_FN_3_O_6_S: C, 56.20; H, 5.33; N, 8.55%, Found, C, 55.09; H, 5.35; N, 8.45%.
Geometry. All s.u.'s (except the s.u. in the dihedral angle between two l.s. planes) are estimated using the full covariance matrix. The cell s.u.'s are taken into account individually in the estimation of s.u.'s in distances, angles and torsion angles; correlations between s.u.'s in cell parameters are only used when they are defined by crystal symmetry. An approximate (isotropic) treatment of cell s.u.'s is used for estimating s.u.'s involving l.s. planes.
Refinement. Refinement of *F*^2^ against ALL reflections. The weighted *R*-factor *wR* and goodness of fit *S* are based on *F*^2^, conventional *R*-factors *R* are based on *F*, with *F* set to zero for negative *F*^2^. The threshold expression of *F*^2^ > 2σ(*F*^2^) is used only for calculating *R*-factors(gt) *etc*. and is not relevant to the choice of reflections for refinement. *R*-factors based on *F*^2^ are statistically about twice as large as those based on *F*, and *R*- factors based on ALL data will be even larger.

### Fractional atomic coordinates and isotropic or equivalent isotropic displacement parameters (Å^2^)

**Table:**

	*x*	*y*	*z*	*U*_iso_*/*U*_eq_	
S1	0.47387 (4)	0.20854 (4)	−0.01227 (2)	0.03209 (16)	
F2	1.00980 (18)	−0.1496 (2)	0.04785 (12)	0.1244 (9)	
O3	0.80071 (13)	0.03367 (15)	0.26846 (9)	0.0512 (4)	
O4	0.70270 (13)	0.38222 (13)	0.31425 (10)	0.0552 (5)	
O5	0.56248 (13)	0.00010 (12)	0.32549 (8)	0.0412 (4)	
O6	0.48854 (13)	0.12205 (13)	−0.06840 (7)	0.0426 (4)	
O7	0.40333 (13)	0.31161 (13)	−0.02950 (8)	0.0434 (4)	
O8	0.91812 (15)	0.39441 (16)	0.11131 (11)	0.0691 (6)	
N9	0.65657 (14)	0.18207 (14)	0.33157 (9)	0.0351 (4)	
N10	0.53633 (14)	0.29687 (14)	0.26198 (9)	0.0354 (4)	
H10	0.5036	0.3604	0.2423	0.042*	
N11	0.42293 (14)	0.13132 (14)	0.04977 (8)	0.0327 (4)	
H11	0.4017	0.0575	0.0414	0.039*	
C12	1.0152 (2)	0.3203 (3)	0.1158 (2)	0.0834 (10)	
H12A	1.0800	0.3661	0.1363	0.125*	
H12B	1.0261	0.2930	0.0679	0.125*	
H12C	1.0056	0.2516	0.1462	0.125*	
C13	0.81799 (19)	0.3448 (2)	0.08321 (13)	0.0463 (5)	
C14	0.7260 (2)	0.4222 (2)	0.07640 (15)	0.0580 (7)	
H14	0.7353	0.5024	0.0916	0.070*	
C15	0.6212 (2)	0.3816 (2)	0.04736 (13)	0.0473 (6)	
H15	0.5595	0.4339	0.0431	0.057*	
C16	0.60774 (17)	0.26262 (18)	0.02452 (10)	0.0338 (4)	
C17	0.69896 (18)	0.18514 (18)	0.03223 (12)	0.0399 (5)	
H17	0.6894	0.1048	0.0174	0.048*	
C18	0.80392 (19)	0.22515 (19)	0.06158 (12)	0.0442 (5)	
H18	0.8651	0.1722	0.0669	0.053*	
C19	0.41262 (16)	0.18727 (16)	0.12110 (10)	0.0287 (4)	
H19	0.4333	0.2731	0.1192	0.034*	
C20	0.29275 (16)	0.17830 (19)	0.13882 (11)	0.0362 (5)	
H20A	0.2691	0.0941	0.1369	0.043*	
H20B	0.2427	0.2230	0.1029	0.043*	
C21	0.28410 (17)	0.2295 (2)	0.21439 (11)	0.0395 (5)	
H21A	0.2994	0.3159	0.2144	0.047*	
H21B	0.2077	0.2182	0.2260	0.047*	
C22	0.36675 (17)	0.16844 (19)	0.27258 (11)	0.0361 (5)	
H22A	0.3624	0.2074	0.3191	0.043*	
H22B	0.3456	0.0840	0.2769	0.043*	
C23	0.48732 (16)	0.17553 (16)	0.25459 (10)	0.0289 (4)	
C24	0.49523 (16)	0.12453 (17)	0.17834 (10)	0.0286 (4)	
H24A	0.5716	0.1353	0.1665	0.034*	
H24B	0.4792	0.0383	0.1780	0.034*	
C25	0.57017 (17)	0.10571 (17)	0.30816 (10)	0.0311 (4)	
C26	0.63708 (18)	0.29892 (17)	0.30243 (11)	0.0367 (5)	
C27	0.76450 (18)	0.1454 (2)	0.37032 (12)	0.0441 (5)	
H27A	0.7544	0.0724	0.3981	0.053*	
H27B	0.7926	0.2087	0.4043	0.053*	
C28	0.84957 (19)	0.1217 (2)	0.31867 (13)	0.0458 (6)	
H28A	0.8655	0.1957	0.2935	0.055*	
H28B	0.9196	0.0916	0.3449	0.055*	
C29	0.85919 (18)	−0.00587 (18)	0.21384 (12)	0.0396 (5)	
C30	0.97395 (19)	0.0047 (2)	0.21463 (14)	0.0531 (6)	
H30	1.0172	0.0442	0.2529	0.064*	
C31	1.0247 (2)	−0.0442 (3)	0.15802 (17)	0.0681 (8)	
H31	1.1024	−0.0383	0.1576	0.082*	
C32	0.9586 (3)	−0.1007 (3)	0.10314 (16)	0.0697 (8)	
C33	0.8452 (2)	−0.1123 (3)	0.10093 (14)	0.0603 (7)	
H33	0.8026	−0.1520	0.0625	0.072*	
C34	0.79498 (19)	−0.0639 (2)	0.15697 (12)	0.0445 (5)	
H34	0.7171	−0.0701	0.1566	0.053*	

### Atomic displacement parameters (Å^2^)

**Table:**

	*U*^11^	*U*^22^	*U*^33^	*U*^12^	*U*^13^	*U*^23^
S1	0.0411 (3)	0.0306 (3)	0.0236 (3)	0.0057 (2)	0.0004 (2)	0.00054 (19)
F2	0.0994 (16)	0.175 (2)	0.1085 (16)	0.0153 (15)	0.0523 (13)	−0.0535 (16)
O3	0.0437 (9)	0.0566 (10)	0.0553 (10)	−0.0118 (7)	0.0141 (8)	−0.0204 (8)
O4	0.0466 (10)	0.0331 (8)	0.0815 (12)	−0.0075 (7)	−0.0092 (8)	−0.0083 (8)
O5	0.0548 (9)	0.0280 (8)	0.0388 (8)	−0.0002 (6)	−0.0021 (7)	0.0041 (6)
O6	0.0604 (10)	0.0421 (8)	0.0248 (7)	0.0048 (7)	0.0039 (7)	−0.0062 (6)
O7	0.0495 (9)	0.0380 (8)	0.0398 (8)	0.0113 (7)	−0.0049 (7)	0.0062 (6)
O8	0.0452 (10)	0.0561 (11)	0.1003 (15)	0.0045 (8)	−0.0131 (10)	−0.0252 (10)
N9	0.0392 (10)	0.0283 (8)	0.0354 (9)	0.0025 (7)	−0.0046 (8)	−0.0064 (7)
N10	0.0425 (10)	0.0230 (8)	0.0383 (10)	0.0022 (7)	−0.0043 (8)	−0.0016 (7)
N11	0.0451 (10)	0.0271 (8)	0.0256 (8)	−0.0028 (7)	0.0033 (7)	−0.0059 (6)
C12	0.0443 (16)	0.0728 (19)	0.126 (3)	0.0066 (14)	−0.0180 (17)	−0.0291 (19)
C13	0.0416 (13)	0.0453 (12)	0.0501 (14)	0.0021 (10)	−0.0018 (10)	−0.0093 (11)
C14	0.0518 (15)	0.0392 (12)	0.0798 (18)	0.0044 (11)	−0.0039 (13)	−0.0195 (12)
C15	0.0448 (13)	0.0353 (12)	0.0599 (15)	0.0106 (10)	−0.0008 (11)	−0.0076 (10)
C16	0.0409 (11)	0.0325 (10)	0.0283 (10)	0.0043 (9)	0.0065 (9)	0.0020 (8)
C17	0.0442 (12)	0.0285 (10)	0.0469 (13)	0.0041 (9)	0.0056 (10)	−0.0014 (9)
C18	0.0410 (12)	0.0382 (12)	0.0526 (14)	0.0107 (10)	0.0034 (10)	0.0002 (10)
C19	0.0367 (11)	0.0249 (9)	0.0242 (9)	−0.0002 (8)	0.0022 (8)	−0.0033 (7)
C20	0.0321 (11)	0.0398 (11)	0.0356 (11)	0.0041 (9)	−0.0001 (9)	0.0002 (9)
C21	0.0332 (11)	0.0441 (12)	0.0424 (12)	0.0063 (9)	0.0088 (9)	−0.0015 (10)
C22	0.0404 (12)	0.0389 (11)	0.0304 (10)	0.0021 (9)	0.0093 (9)	−0.0013 (9)
C23	0.0355 (11)	0.0234 (9)	0.0273 (10)	0.0003 (8)	0.0023 (8)	−0.0019 (7)
C24	0.0305 (10)	0.0274 (9)	0.0276 (10)	0.0017 (8)	0.0028 (8)	−0.0023 (7)
C25	0.0402 (11)	0.0288 (10)	0.0242 (10)	0.0021 (8)	0.0034 (8)	−0.0042 (8)
C26	0.0397 (12)	0.0286 (10)	0.0408 (12)	0.0019 (9)	0.0013 (9)	−0.0073 (8)
C27	0.0452 (13)	0.0442 (12)	0.0389 (12)	0.0065 (10)	−0.0103 (10)	−0.0079 (10)
C28	0.0397 (12)	0.0417 (12)	0.0533 (14)	−0.0008 (10)	−0.0052 (10)	−0.0076 (10)
C29	0.0412 (12)	0.0351 (11)	0.0434 (12)	0.0014 (9)	0.0086 (10)	0.0045 (9)
C30	0.0383 (13)	0.0600 (15)	0.0608 (15)	−0.0006 (11)	0.0054 (11)	0.0004 (12)
C31	0.0419 (14)	0.082 (2)	0.084 (2)	0.0089 (14)	0.0229 (14)	0.0029 (17)
C32	0.0637 (18)	0.086 (2)	0.0642 (18)	0.0148 (15)	0.0266 (15)	−0.0129 (15)
C33	0.0612 (17)	0.0694 (17)	0.0505 (15)	0.0038 (13)	0.0083 (13)	−0.0111 (13)
C34	0.0404 (12)	0.0468 (13)	0.0464 (13)	0.0008 (10)	0.0065 (10)	0.0011 (10)

### Geometric parameters (Å, °)

**Table:**

S1—O7	1.4258 (15)	C19—C20	1.510 (3)
S1—O6	1.4370 (15)	C19—C24	1.519 (3)
S1—N11	1.6092 (17)	C19—H19	0.9800
S1—C16	1.759 (2)	C20—C21	1.524 (3)
F2—C32	1.367 (3)	C20—H20A	0.9700
O3—C29	1.370 (3)	C20—H20B	0.9700
O3—C28	1.417 (3)	C21—C22	1.522 (3)
O4—C26	1.209 (2)	C21—H21A	0.9700
O5—C25	1.214 (2)	C21—H21B	0.9700
O8—C13	1.356 (3)	C22—C23	1.518 (3)
O8—C12	1.411 (3)	C22—H22A	0.9700
N9—C25	1.358 (3)	C22—H22B	0.9700
N9—C26	1.405 (3)	C23—C25	1.518 (3)
N9—C27	1.449 (3)	C23—C24	1.533 (3)
N10—C26	1.333 (3)	C24—H24A	0.9700
N10—C23	1.459 (2)	C24—H24B	0.9700
N10—H10	0.8600	C27—C28	1.502 (3)
N11—C19	1.476 (2)	C27—H27A	0.9700
N11—H11	0.8600	C27—H27B	0.9700
C12—H12A	0.9600	C28—H28A	0.9700
C12—H12B	0.9600	C28—H28B	0.9700
C12—H12C	0.9600	C29—C30	1.372 (3)
C13—C18	1.383 (3)	C29—C34	1.378 (3)
C13—C14	1.383 (3)	C30—C31	1.384 (4)
C14—C15	1.372 (3)	C30—H30	0.9300
C14—H14	0.9300	C31—C32	1.356 (4)
C15—C16	1.382 (3)	C31—H31	0.9300
C15—H15	0.9300	C32—C33	1.354 (4)
C16—C17	1.376 (3)	C33—C34	1.370 (3)
C17—C18	1.373 (3)	C33—H33	0.9300
C17—H17	0.9300	C34—H34	0.9300
C18—H18	0.9300		
			
O7—S1—O6	119.32 (9)	C20—C21—H21B	109.2
O7—S1—N11	108.41 (9)	H21A—C21—H21B	107.9
O6—S1—N11	104.85 (9)	C23—C22—C21	111.63 (16)
O7—S1—C16	107.30 (10)	C23—C22—H22A	109.3
O6—S1—C16	108.50 (10)	C21—C22—H22A	109.3
N11—S1—C16	108.01 (9)	C23—C22—H22B	109.3
C29—O3—C28	119.76 (17)	C21—C22—H22B	109.3
C13—O8—C12	117.8 (2)	H22A—C22—H22B	108.0
C25—N9—C26	111.34 (16)	N10—C23—C25	100.59 (15)
C25—N9—C27	125.11 (17)	N10—C23—C22	113.94 (16)
C26—N9—C27	122.64 (18)	C25—C23—C22	112.77 (16)
C26—N10—C23	112.96 (15)	N10—C23—C24	110.60 (15)
C26—N10—H10	123.5	C25—C23—C24	107.72 (15)
C23—N10—H10	123.5	C22—C23—C24	110.69 (16)
C19—N11—S1	119.92 (13)	C19—C24—C23	111.41 (15)
C19—N11—H11	120.0	C19—C24—H24A	109.3
S1—N11—H11	120.0	C23—C24—H24A	109.3
O8—C12—H12A	109.5	C19—C24—H24B	109.3
O8—C12—H12B	109.5	C23—C24—H24B	109.3
H12A—C12—H12B	109.5	H24A—C24—H24B	108.0
O8—C12—H12C	109.5	O5—C25—N9	126.13 (18)
H12A—C12—H12C	109.5	O5—C25—C23	126.45 (18)
H12B—C12—H12C	109.5	N9—C25—C23	107.39 (16)
O8—C13—C18	124.4 (2)	O4—C26—N10	129.17 (19)
O8—C13—C14	115.9 (2)	O4—C26—N9	123.75 (19)
C18—C13—C14	119.8 (2)	N10—C26—N9	107.08 (17)
C15—C14—C13	120.5 (2)	N9—C27—C28	111.25 (18)
C15—C14—H14	119.7	N9—C27—H27A	109.4
C13—C14—H14	119.7	C28—C27—H27A	109.4
C14—C15—C16	119.6 (2)	N9—C27—H27B	109.4
C14—C15—H15	120.2	C28—C27—H27B	109.4
C16—C15—H15	120.2	H27A—C27—H27B	108.0
C17—C16—C15	119.9 (2)	O3—C28—C27	106.35 (18)
C17—C16—S1	119.78 (16)	O3—C28—H28A	110.5
C15—C16—S1	120.32 (16)	C27—C28—H28A	110.5
C18—C17—C16	120.7 (2)	O3—C28—H28B	110.5
C18—C17—H17	119.6	C27—C28—H28B	110.5
C16—C17—H17	119.6	H28A—C28—H28B	108.7
C17—C18—C13	119.5 (2)	O3—C29—C30	124.4 (2)
C17—C18—H18	120.3	O3—C29—C34	115.19 (19)
C13—C18—H18	120.3	C30—C29—C34	120.3 (2)
N11—C19—C20	110.81 (15)	C29—C30—C31	119.4 (2)
N11—C19—C24	108.26 (15)	C29—C30—H30	120.3
C20—C19—C24	111.53 (16)	C31—C30—H30	120.3
N11—C19—H19	108.7	C32—C31—C30	118.5 (2)
C20—C19—H19	108.7	C32—C31—H31	120.7
C24—C19—H19	108.7	C30—C31—H31	120.7
C19—C20—C21	110.55 (16)	C33—C32—C31	123.3 (2)
C19—C20—H20A	109.5	C33—C32—F2	118.6 (3)
C21—C20—H20A	109.5	C31—C32—F2	118.0 (3)
C19—C20—H20B	109.5	C32—C33—C34	118.1 (3)
C21—C20—H20B	109.5	C32—C33—H33	121.0
H20A—C20—H20B	108.1	C34—C33—H33	121.0
C22—C21—C20	111.87 (17)	C33—C34—C29	120.3 (2)
C22—C21—H21A	109.2	C33—C34—H34	119.8
C20—C21—H21A	109.2	C29—C34—H34	119.8
C22—C21—H21B	109.2		
			
O7—S1—N11—C19	−60.56 (16)	N10—C23—C24—C19	−72.4 (2)
O6—S1—N11—C19	170.97 (14)	C25—C23—C24—C19	178.57 (15)
C16—S1—N11—C19	55.41 (16)	C22—C23—C24—C19	54.9 (2)
C12—O8—C13—C18	2.9 (4)	C26—N9—C25—O5	−178.3 (2)
C12—O8—C13—C14	−176.9 (3)	C27—N9—C25—O5	12.4 (3)
O8—C13—C14—C15	178.8 (2)	C26—N9—C25—C23	3.5 (2)
C18—C13—C14—C15	−1.0 (4)	C27—N9—C25—C23	−165.77 (18)
C13—C14—C15—C16	−0.3 (4)	N10—C23—C25—O5	175.12 (19)
C14—C15—C16—C17	1.2 (3)	C22—C23—C25—O5	53.4 (3)
C14—C15—C16—S1	179.56 (19)	C24—C23—C25—O5	−69.1 (2)
O7—S1—C16—C17	−166.69 (16)	N10—C23—C25—N9	−6.72 (19)
O6—S1—C16—C17	−36.52 (19)	C22—C23—C25—N9	−128.47 (17)
N11—S1—C16—C17	76.62 (18)	C24—C23—C25—N9	109.10 (17)
O7—S1—C16—C15	14.9 (2)	C23—N10—C26—O4	173.8 (2)
O6—S1—C16—C15	145.07 (18)	C23—N10—C26—N9	−6.5 (2)
N11—S1—C16—C15	−101.79 (19)	C25—N9—C26—O4	−178.7 (2)
C15—C16—C17—C18	−0.9 (3)	C27—N9—C26—O4	−9.1 (3)
S1—C16—C17—C18	−179.27 (17)	C25—N9—C26—N10	1.6 (2)
C16—C17—C18—C13	−0.4 (3)	C27—N9—C26—N10	171.25 (17)
O8—C13—C18—C17	−178.5 (2)	C25—N9—C27—C28	92.0 (2)
C14—C13—C18—C17	1.3 (4)	C26—N9—C27—C28	−76.1 (2)
S1—N11—C19—C20	125.10 (15)	C29—O3—C28—C27	179.60 (18)
S1—N11—C19—C24	−112.29 (16)	N9—C27—C28—O3	−55.9 (2)
N11—C19—C20—C21	176.69 (16)	C28—O3—C29—C30	20.0 (3)
C24—C19—C20—C21	56.0 (2)	C28—O3—C29—C34	−162.7 (2)
C19—C20—C21—C22	−55.3 (2)	O3—C29—C30—C31	176.7 (2)
C20—C21—C22—C23	54.9 (2)	C34—C29—C30—C31	−0.4 (4)
C26—N10—C23—C25	8.2 (2)	C29—C30—C31—C32	0.3 (4)
C26—N10—C23—C22	129.10 (19)	C30—C31—C32—C33	−0.2 (5)
C26—N10—C23—C24	−105.47 (19)	C30—C31—C32—F2	−179.4 (3)
C21—C22—C23—N10	71.3 (2)	C31—C32—C33—C34	0.3 (5)
C21—C22—C23—C25	−174.89 (16)	F2—C32—C33—C34	179.4 (3)
C21—C22—C23—C24	−54.1 (2)	C32—C33—C34—C29	−0.4 (4)
N11—C19—C24—C23	−178.48 (15)	O3—C29—C34—C33	−176.9 (2)
C20—C19—C24—C23	−56.3 (2)	C30—C29—C34—C33	0.5 (3)

### Hydrogen-bond geometry (Å, °)

**Table:**

*D*—H···*A*	*D*—H	H···*A*	*D*···*A*	*D*—H···*A*
N10—H10···O5^i^	0.86	2.08	2.924 (2)	168.
N11—H11···O6^ii^	0.86	2.39	2.991 (3)	127.
C20—H20A···O4^iii^	0.97	2.51	3.377 (3)	148.
C31—H31···O4^iv^	0.93	2.48	3.327 (3)	152.

Symmetry codes: (i) −*x*+1, *y*+1/2, −*z*+1/2; (ii) −*x*+1, −*y*, −*z*; (iii) −*x*+1, *y*−1/2, −*z*+1/2; (iv) −*x*+2, *y*−1/2, −*z*+1/2.
